# Clinicopathologic Features Associated with Survival for Immune Checkpoint Blockade in Patients with Metastatic Anal Cancer

**DOI:** 10.3390/cancers17060937

**Published:** 2025-03-10

**Authors:** Arjun S. Peddireddy, Ryan Huey, Robert A. Wolff, Kangyu Lin, Jocelyn Mitchell, Lisa Scofield, Sophia Jacob, Diem V. Nguyen, Jane Rogers, Shaelynn Portier, Wai Chin Foo, Cathy Eng, Van K. Morris

**Affiliations:** 1 McGovern Medical School at UT Health, Houston, TX 77030, USA; arjun.s.peddireddy@uth.tmc.edu; 2 Department of Gastrointestinal Medical Oncology, The University of Texas MD Anderson Cancer Center, Houston, TX 77030, USA; rwhuey@mdanderson.org (R.H.); rwolff@mdanderson.org (R.A.W.); klin1@mdanderson.org (K.L.); jmrausch@mdanderson.org (J.M.); lclayton@mdanderson.org (L.S.); sejacob1@mdanderson.org (S.J.); smportier@mdanderson.org (S.P.); 3 Department of Pharmacy, The University of Texas MD Anderson Cancer Center, Houston, TX 77030, USA; dvnguyen1@mdanderson.org (D.V.N.); jerogers@mdanderson.org (J.R.); 4 Department of Pathology, The University of Texas MD Anderson Cancer Center, Houston, TX 77030, USA; wfoo@mdanderson.org; 5 Vanderbilt-Ingram Cancer Center, Nashville, TN 37232, USA; cathy.eng@vumc.org

**Keywords:** immunotherapy, anal cancer, cancer survival

## Abstract

**Background/Objectives**: Anal cancer is a rare malignancy with limited treatment options. Immune checkpoint inhibitors have shown benefits in some patients with metastatic disease, but predictive factors for immunotherapy response remain undefined. This study retrospectively evaluated clinical and pathological features associated with survival outcomes in metastatic anal cancer treated with immunotherapy. **Methods**: Data from 105 patients with metastatic anal cancer were analyzed. Kaplan–Meier analysis was used to estimate progression-free survival (PFS) and overall survival (OS), with subgroup comparisons utilizing the Mantel–Cox test. Associations between survival and clinicopathologic features were assessed with Fisher’s exact test. **Results**: Of the patients, 69 (65.7%) received immunotherapy during the first three treatment lines. With a median follow-up of 23.2 months, the median PFS for first-, second-, and third-line systemic therapies was 7.2, 3.7, and 4.7 months, respectively (χ^2^ = 14.2; *p* < 0.001). In the treatment-refractory setting, median PFS was similar for immunotherapy and chemotherapy: 3.6 months (95% CI, 2.3–4.9) vs. 4.4 months (95% CI, 3.8–5.0), respectively (HR 0.89, 95% CI 0.60–1.3; *p* = 0.52). Among patients treated with immunotherapy, patients with lymph node-only metastases had significantly prolonged PFS compared to patients with visceral organ involvement (11.3 vs. 3.1 months; HR 0.49, 95% CI 0.21–0.74; *p* = 0.03). **Conclusions**: Patients with lymph node-only metastatic anal cancer experienced significantly prolonged PFS with immunotherapy relative to those with involvement of other distant organs, highlighting a distinct subgroup of patients who may benefit from immunotherapy. We also contextualize PFS outcomes across treatment lines for metastatic anal cancer, which can be applied towards the design of future immunotherapy clinical trials.

## Simple Summary

In addition to cytotoxic chemotherapy, strategies for the treatment of metastatic anal cancer have evolved to include immune checkpoint inhibitors. However, biomarkers that can predict immunotherapy treatment outcomes are limited and the aim of this study is to identify patient- and disease-related characteristics that predict outcomes of immunotherapy in the treatment of metastatic anal cancer. This retrospective study analyzed 105 patients with unresectable or metastatic anal cancer, using Kaplan–Meier analyses to assess progression-free and overall survival across sequential lines of treatment. These findings highlight one of the first analyses to characterize progression-free survival across treatment lines for metastatic anal cancer, showing that the metastatic pattern can influence response to immunotherapy. Lymph node-only metastases were identified as a potential positive predictive factor for favorable outcomes, but this research also points out the need for future trials that can refine personalized strategies for the treatment of metastatic anal cancer.

## 1. Introduction

Over the past decade, there has been an estimated 2.2% annual rise in newly diagnosed cases of anal cancer [1], with more than 10,000 new cases expected in the United States in 2024 [2]. Anal cancer is primarily categorized into two histological subtypes: squamous cell carcinoma, which accounts for the vast majority of cases, and adenocarcinoma, a rarer phenotype [3]. Approximately 90% of squamous cell carcinomas of the anus are associated with human papillomavirus (HPV) infection, with high-risk variants such as HPV16 being detected in 83% to 85% of these tumors [4,5]. The progression of HPV infection to cancer likely occurs over years to decades, requiring the virus to evade the human immune system [6]. This evasion is achieved through mechanisms such as the downregulation of proinflammatory cytokines, such as interferons, and the upregulation of immune checkpoint proteins like programmed cell death ligand 1 (PD-L1). These processes, combined with the inhibition of tumor suppressor genes such as P53 and pRB, drive the oncogenesis caused by HPV [6,7]. Of great concern, the relative proportion of metastatic presentation for anal cancer has increased, accompanied by a 4% rise in annual mortality during this time [8]. For the 10% of all anal cancer patients who are diagnosed at the metastatic stage and the 10–20% of chemoradiotherapy-treated patients who progress to metastatic anal cancer, the prognosis remains poor with a 5-year relative survival rate of 30% [9]. The most common sites of distant metastases include the liver, lung, and extra-pelvic distant lymph nodes [9,10].

While the standard paradigm of concurrent chemoradiation has not changed over the past five decades for patients with localized anal cancer [11,12], the landscape of options available for the treatment of metastatic disease has rapidly evolved over the past 10 years. For patients with diffuse metastatic disease or locally unresectable anal cancer, systemic treatment for incurable disease often centers around cytotoxic chemotherapy. Doublet chemotherapy with carboplatin and paclitaxel demonstrated improved overall survival relative to cisplatin/5-fluorouracil for frontline treatment of metastatic anal cancer [13], and other studies have shown similar overall response rates between the two chemotherapy combinations [14]. Triplet cytotoxic chemotherapy with docetaxel, cisplatin, and 5-fluorouracil has also shown remarkable efficacy as initial treatment for metastatic anal cancer for those robust enough to tolerate the toxicity [15,16].

Beyond chemotherapy, the treatment landscape of metastatic/unresectable anal cancer has broadened with the advent of anti-programmed death-(ligand) 1 [anti-PD-(L)1] antibodies, with responses reported in 11–24% of cases with treatment-refractory incurable anal cancer [17,18,19,20,21]. Molecular biomarkers predictive for benefit from immunotherapy have not been robustly validated for patients with metastatic anal cancer. For instance, microsatellite instability-high status, a biomarker predictive of response to immunotherapy in colorectal adenocarcinoma [22,23,24,25], is uncommon in patients with anal cancer [26] and not routinely tested.

To our knowledge, there has not been reporting of outcomes assessing clinicopathologic features for patients with metastatic anal cancer following treatment with immunotherapy. To that end, we retrospectively profiled survival across sequential lines of chemotherapy or immunotherapy for 105 patients with unresectable and/or metastatic anal cancer at our institution.

## 2. Materials and Methods

### 2.1. Selection of Patients with Incurable Anal Cancer

Patients selected for this retrospective study included those who were evaluated and treated at MD Anderson Cancer Center for the diagnosis of squamous cell carcinoma of the anus or rectum between June 2014 and November 2023. No patients with adenocarcinoma of the anus or rectum were included in this analysis. All included study participants had consented to participate in an Institutional Review Board-approved observational study which allowed for analysis of clinical, pathologic, and outcome data of anal cancer subsequently obtained from the electronic medical record. Only patients with incurable, unresectable, and/or metastatic anal cancer were included; those with locoregional anal cancer treated definitively with chemoradiation were not assessed. The archival tumor tissue was evaluated for HPV positivity according to the detection of p16 protein expression by immunohistochemistry and/or the presence of HPV DNA by in situ hybridization. Features of the study population were characterized using descriptive statistics. Differences in mean values for subgroups of interest were compared with Student’s *t*-test. Associations between selected clinical or pathologic characteristics and metastatic disease distribution were assessed using Fisher’s exact *t*-test.

### 2.2. Survival Analysis

Metastatic survival (OS) was calculated as the time between date of diagnosis of unresectable anal cancer and date of death or date of last follow-up (whichever came first). Progression-free survival (PFS) was measured as the time between the date of initiation of a line of therapy and the date of clinical or radiographic progression—as determined according to the treating oncologist—whichever came first. For each of the first, second, and third lines of therapy (if applicable), progression-free survival was calculated for each patient as PFS1, PFS2, and PFS3, respectively. Median survival outcomes, along with the associated 95% confidence interval (CI), were estimated according to the Kaplan–Meier method. Hazard ratios (HRs) were calculated using the log-rank method, and median survival outcomes were compared using a Mantel–Cox test. A two-sided *p*-value less than 0.05 was considered statistically significant in evaluating these comparisons.

## 3. Results

### 3.1. Clinicopathologic Features and Treatment Selection

Clinical and pathologic features for 105 patients with incurable, metastatic anal cancer treated at our institution are summarized in Table 1. The median age at the time of diagnosis of anal cancer was 59 years (range, 42–81). The majority (80%) of patients identified as female, and 95% were not living with human immunodeficiency virus (HIV). For the 52 patients for whom Eastern Cooperative Oncology Group performance status (ECOG PS) was documented at the time of metastatic diagnosis, 58% of patients had a performance status of 0, 38% had a performance status of 1, and 4% had a performance status of 2. Archival tissue was available in 78/105 cases, and among these, 97% were HPV-positive tumors. The number of distant metastatic sites varied, with 47 (45%) patients developing metastases in a single organ, 31 (30%) patients developing metastases in two organs, 23 (22%) patients developing metastases in three organs, and 4 (4%) patients developing metastases in four or more organs. The most common sites of metastasis included the liver (47%), lung (38%), and lymph nodes (27%), while 24% of patients had unresectable/locally recurrent disease. Less frequently, metastases were observed in bone (11%) and the peritoneum (8%). The occurrence of metastatic disease at initial presentation occurred in 64 (61%) patients, whereas metachronous development of distant metastases happened in 39% of cases. Among patients with metachronous disease development, there was no difference in time to development of distant metastases (17.0 months vs. 15.0 months, respectively; HR 0.65, 95% CI 0.30–1.30; *p* = 0.35) for lymph node only vs. visceral organ involvement, respectively. Prior radiation to the primary anal cancer had been administered in 77 (73%) patients.

The median number of lines of systemic treatment for metastatic anal cancer was 2 (range, 1–6). Patients with lymph node-only metastases received a median of two therapeutic lines of treatment (range, 1–5), compared to patients with visceral organ metastases who also received a median of two lines of treatment (range 1–6). Per Table 2, most patients received doublet cytotoxic chemotherapy as frontline treatment, with a platinum/taxane combination as the most common regimen. Only five patients were administered upfront triplet cytotoxic chemotherapy with docetaxel, cisplatin, and 5-fluorouracil. In the first-line setting, nine patients were deemed inappropriate for cytotoxic chemotherapy and were offered an anti-PD-1 antibody as monotherapy instead. In five other cases, eligible patients opted to participate in immunotherapy combination trials—either the anti-PD-L1 antibody atezolizumab with the anti-vascular endothelial growth factor antibody bevacizumab, or the anti-PD-L1 antibody durvalumab with the therapeutic HPV-16/18 E6/E7 DNA vaccine MEDI-0457—as frontline treatment.

Immune checkpoint blockade was the most common therapeutic approach for the 74 patients with metastatic anal cancer receiving systemic treatment in the second-line setting (46/74). Most patients (35/46, 74%) here were treated with anti-PD-1 antibodies as monotherapy. There were 44 patients who received third-line systemic therapy, of which 9 (20%) received immunotherapy. Only 13 (12%) out of the entire cohort received more than three lines of treatment.

### 3.2. Survival Outcomes

**Figure 1:**
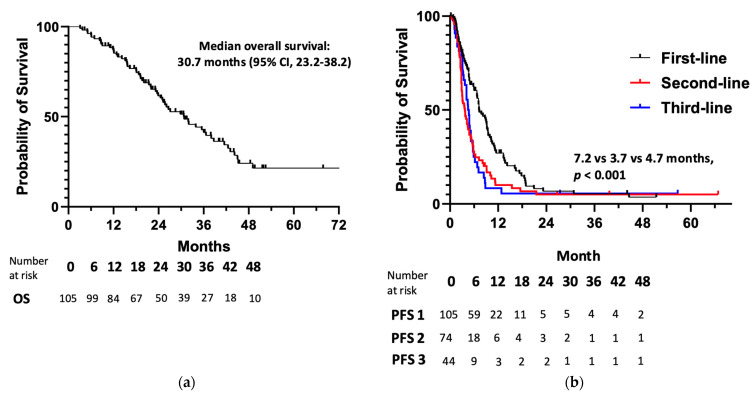
(**a**) Overall survival and (**b**) progression-free survival for lines 1 (PFS 1), 2 (PFS 2), and 3 (PFS 3) of systemic therapy for patients with metastatic anal cancer, where PFS 1 represents progression-free survival after a patient’s first line of therapy, PFS 2 represents progression-free survival after a patient’s second line of therapy, and PFS 3 represents progression-free survival after a patient’s third line of therapy.

After a median follow-up of 23.2 months, the median OS (Figure 1a) for the 105 patients with metastatic anal cancer was 30.7 months (95% CI, 23.2–38.2). The proportion of patients alive at 1, 2, 3, 4, and 5 years after initial presentation of metastatic disease was 86%, 62%, 41%, 24%, and 21%, respectively.

**Figure 2:**
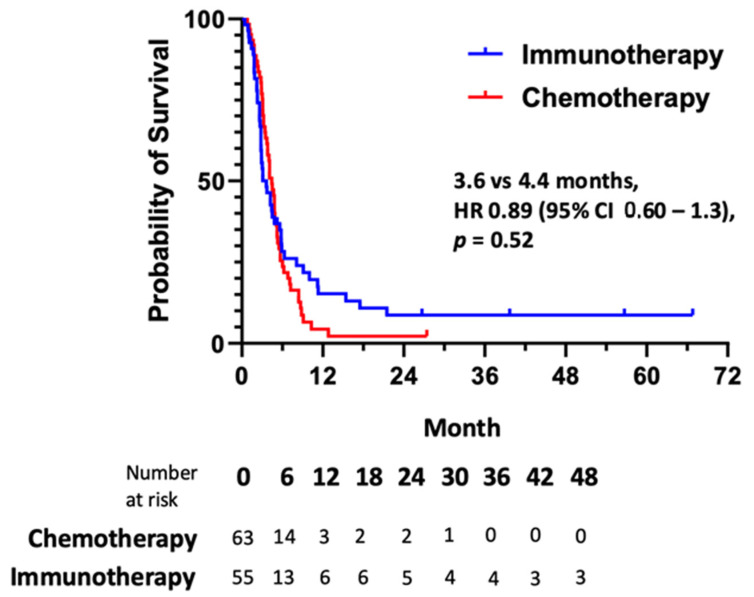
Progression-free survival according to class of systemic therapy utilized for treatment-refractory advanced anal cancer.

**Figure A1:**
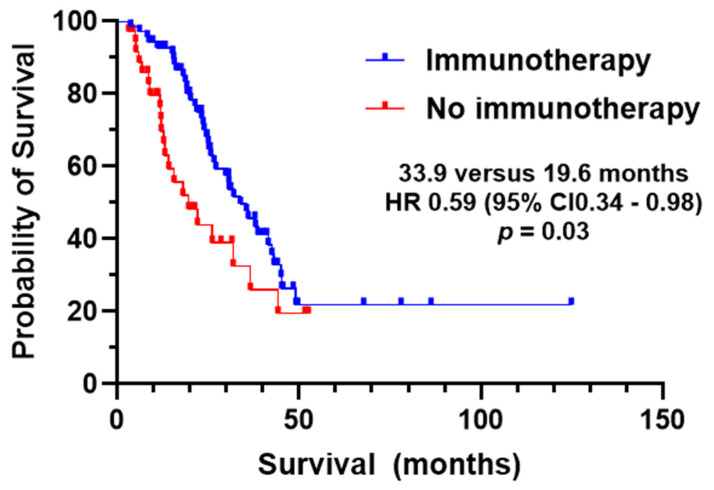
Overall survival according to exposure to immunotherapy.

As seen in Figure 1b, differences in median PFS were observed for sequential lines of therapy (χ2 = 14.2; *p* < 0.001), with first-line systemic treatment demonstrating the longest median PFS of 7.2 months (95% CI, 5.5–8.9). PFS outcomes were shorter in the second and third lines at 3.7 months (95% CI, 2.8–4.6) and 4.7 months (95% CI, 3.9–5.5), respectively. Because immunotherapy is listed as a recommended treatment option in the United States in the treatment-refractory setting by the NCCN Guidelines for Anal Cancer, we compared outcomes according to treatment type (i.e., cytotoxic chemotherapy or immunotherapy) in the combined second- and third-line settings. As seen in Figure 2, there was no difference in median PFS (HR 0.89, 95% CI 0.60–1.3; *p* = 0.52) between immunotherapy (3.6 months, 95% CI 2.3–4.9) and chemotherapy (4.4 months, 95% CI 3.8–5.0), and these groups had no differences in their clinical and pathological features (Table A1). However, we did observe prolonged median OS for patients who received immunotherapy as part of their treatment when compared to those who received cytotoxic chemotherapy only (33.9 months versus 19.6 months, HR 0.59, 95% CI 0.34–0.98; *p* = 0.03; Figure A1).

For the 69 patients who received immunotherapy in the first-, second-, or third-line settings, there were 9 (13%) who had only lymph nodes (outside the pelvis) as their lone site of metastatic involvement. The remaining 60 patients had developed metastatic lesions in visceral organs, including 31 (45%) who had liver metastases, 29 (42%) who had lung metastases, and 5 (7%) who had peritoneal metastases, with 51 patients in this subgroup developing lesions in multiple organs. There were no differences in clinicopathologic features between these lymph node-only and visceral metastasis subgroups of patients with respect to age (mean 59.7 versus 58.5 years, *p* = 0.28), female gender (odds ratio (OR) 6.5, 95% CI 0.36–120; *p* = 0.21), HPV-positive tumor (OR 0.57, 95% CI 0.02–15; *p* = 0.74), prior receipt of radiation (OR 0.72, 95% CI 0.16–3.3; *p* = 0.68), or development of metachronous distant metastases (OR 0.65, 95% CI 0.16–27; *p* = 0.56). Of the nine patients with lymph node-only involvement who received immunotherapy, three featured supradiaphragmatic lymphadenopathy, and the remaining six had diffuse retroperitoneal, paraaortic, retrocaval, and/or aortocaval lymph node involvement that was outside the pelvic radiation field incorporated in the treatment of locoregional anal cancer.

**Figure 3:**
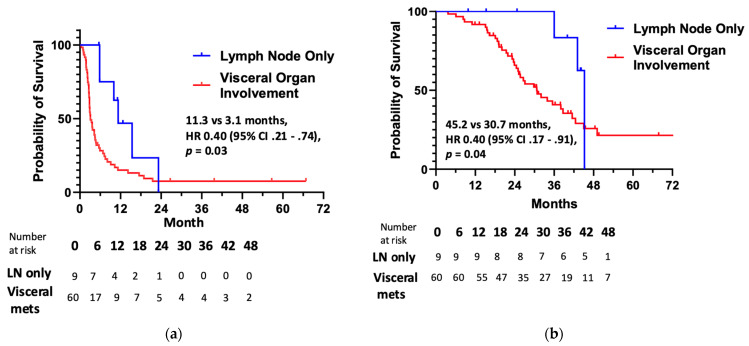
(**a**) Progression-free survival and (**b**) overall survival for immunotherapy according to distribution of metastatic disease.

**Figure A2:**
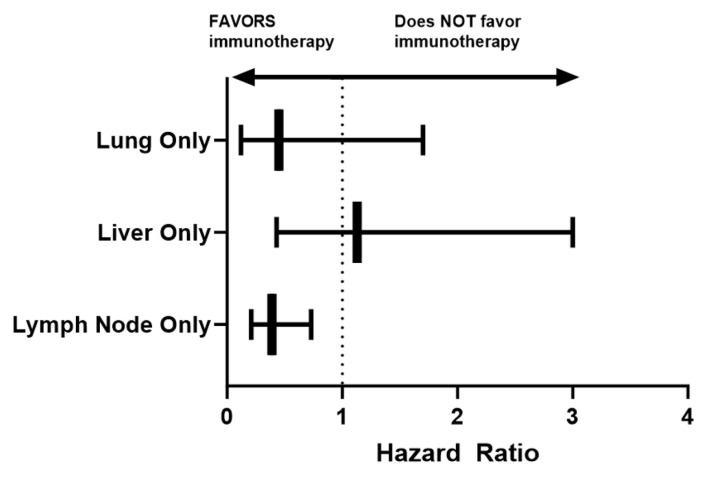
Comparison of hazard ratios of progression-free survival among patients with metastatic anal cancer treated with immunotherapy for lung-only metastases, liver-only metastases, and lymph node-only metastases.

The median PFS with immunotherapy was prolonged for patients with lymph node-only metastases (HR 0.49, 95% CI 0.21–0.74; *p* = 0.03) relative to those with visceral organ involvement (Figure 3a)—11.3 months (95% CI, 6.2–16.3) versus 3.1 months (95% CI, 2.3–3.9). For the lymph node-only cohort, rates of 6-, 12-, and 18-month PFS with immunotherapy were 75%, 62%, and 23%, respectively. Alternatively, the same PFS rates were 30%, 15%, and 11% for those with visceral metastatic anal cancer who received immune checkpoint blockade. There was no difference in median PFS for the five patients with liver-only metastases or the four patients with lung-only metastases who received immunotherapy (Figure A2).

While no clinicopathologic factors were associated with improved OS for the entire population of patients (regardless of immunotherapy treatment) in this retrospective study (Table A2), we did identify favorable prognostic features among the cohort of patients who were administered immunotherapy. As seen in Figure 3b, the median OS was greater for patients with lymph node-only metastases who received immunotherapy (45.2 months versus 30.7 months; HR 0.40, 95% CI 0.17–0.91; *p* = 0.04). An ECOG PS of 0 was associated with improved OS among patients receiving immunotherapy as well Table A3). Additionally, there was a trend towards prolonged OS in patients with lymph node-only metastases across the entire cohort of metastatic anal cancer patients in our analysis, regardless of whether or not they received immunotherapy (44.2 months versus 26.1 months; HR 0.59, 95% CI 0.24–1.47; *p* = 0.12).

## 4. Discussion

To our knowledge, we present here the first series to characterize PFS across sequential lines of systemic therapy for patients with metastatic anal cancer. That multiple lines are even available as options is reflective of the evolving landscape of treatment possibilities for this patient population over the past decade, with the advent of new cytotoxic combinations and of immunotherapy as proven effective therapeutics. It is also noteworthy that patients with metastatic anal cancer who were treated with immunotherapy experienced a longer median OS than those who received only cytotoxic chemotherapy. Given recommendations for immune checkpoint blockade as treatment for metastatic anal cancer as listed in the NCCN Guidelines, these findings lend further support to the incorporation of these therapies into clinical care for patients with this disease.

It is notable that in our series, median PFS in the first line of treatment was significantly longer than that of subsequent lines of systemic therapy. Here, the majority of patients were administered doublet cytotoxic chemotherapy, as fewer than 5% of our study cohort received triplet cytotoxic chemotherapy with taxane, fluoropyrimidine, and platinum agents. Although two- versus three-drug regimens have yet to be compared head-to-head prospectively for patients with metastatic anal cancer, an overall response rate of 87% for the combination of docetaxel, cisplatin, and 5-fluorouracil was reported in the frontline setting for patients with previously untreated metastatic anal cancer [15], nominally higher than those reported for carboplatin/paclitaxel (57%) or 5-fluorouracil/cisplatin (59%) in the same setting. It is unlikely that the progression-free survival benefit that we observed for frontline treatment of metastatic anal cancer in this retrospective cohort then was due to a disproportionate use of a three-drug cytotoxic regimen otherwise not evenly distributed in subsequent treatments after initial progression.

That PFS was shorter in the second- and third-line settings is suggestive of an adaptation of tumor biology that renders metastatic anal cancer less susceptible to sequential lines of systemic treatment. We did not include fourth or higher lines of treatment in our analysis because only a small fraction of patients received such therapies and because most patients were offered off-label therapies as part of experimental clinical trials. Our findings do however provide context for median PFS metrics in the second- and third-line settings against which survival outcomes for treatment-refractory, unresectable, and/or metastatic anal cancer can be compared in future clinical trials.

We did not observe any difference in median PFS with the use of immune checkpoint blockade (3.6 months) relative to cytotoxic chemotherapy (4.4 months) in the treatment-refractory setting. No prospective study has compared these approaches directly in patients with metastatic anal cancer, and our data suggest clinical equipoise between these two options in terms of survival outcomes. Of note, we did include patients who received combination immunotherapy as part of clinical trials in our analysis. In support of this decision, treatment with atezolizumab and bevacizumab [27] or with durvalumab and MEDI-0457 [28] was associated with similar overall response rates and median PFS as had been detailed in separate single-arm trials of anti-PD-(L)1 therapies alone. Therefore, our analysis of outcomes using immunotherapy predominantly includes the anti-PD-(L)1 component common to all of these administered treatments.

A novel finding reported here is that PFS with immunotherapy is significantly prolonged in patients with metastatic anal cancer according to the pattern of metastatic spread. Patients with lymph node-only distant metastases featured longer median PFS (11.3 months) than those with metastatic involvement of other organs (3.1 months). Similarly, lymph node-only metastatic involvement was prognostic for increased overall survival (45.2 versus 30.7 months) in patients receiving immunotherapy. While the overall prevalence of lymph node-only metastatic involvement was relatively low at 13% among all patients with incurable anal cancer in our review, it is important to note that the overall response rate of immune checkpoint blockade with immunotherapy is similarly low (11–24%) [17,18,19,20,21] and that this may provide important insight into the infrequent subgroup of patients with metastatic anal cancer who derive clinical benefit from immunotherapy. The notion that the site of organ involvement correlates with efficacy for immunotherapy has been reported in other solid tumors like microsatellite-stable metastatic colorectal cancer, whereby the use of a targeted therapy plus immunotherapy combination was linked to response rates of 19.5% versus 0% for patients without or with liver metastases, respectively [29]. Molecular biomarkers like PD-(L)1 expression and tumor mutation burden have not been definitively validated as predictive for improved clinical outcomes for patients with metastatic anal cancer who received immunotherapy. Our data provide initial insight into identifying who may or may not benefit from these agents by connotating a lymph node-only pattern of metastatic spread as a favorable factor associated with improved survival outcomes.

Our findings are limited by the retrospective nature of this work as a single-institution study. Indeed, many of our patients harbored a robust performance status that permitted routine travel to our site for their care, which infers an ascertainment bias in overestimating survival outcomes for our cohort relative to the general population of patients with metastatic anal cancer. Furthermore, we acknowledge the limitation of PD-L1 testing not occurring routinely in this described patient analysis. Future efforts to evaluate PD-L1 expression in relation to the site of distant metastatic organ involvement for metastatic anal cancer would be of interest. However, to date, prospective immunotherapy trials have enrolled small numbers of participants as single-arm studies for this rare malignancy in the treatment-refractory setting, which unfortunately included few responders per study and limits the ability to draw definitively needed insight into patterns of treatment benefit. The large phase III POD1UM-303 recently reported improvement in progression-free survival with the addition of retifanlimab immunotherapy to carboplatin and paclitaxel in the frontline setting [30]. However, the interaction between the modality of treatment (i.e., chemotherapy versus immunotherapy) and the site of metastatic organ involvement remains unclear. By establishing a larger institutional cohort of patients, the majority of whom received immunotherapy, we were able to overcome historical challenges in characterization of outcomes for a less common disease like anal cancer.

## 5. Conclusions

Immunotherapy demonstrates similar survival outcomes in comparison to the more utilized cytotoxic chemotherapy options for treatment-refractory metastatic anal cancer. That first-line median PFS was prolonged relative to that of subsequent lines of therapy highlights the need for additional effective treatment options for patients with metastatic anal cancer. We acknowledge the low number of patients with lymph node-only disease in our retrospective cohort who received immunotherapy. However, this is an orphan malignancy, and our findings here are hypothesis-generating and should be considered for validation more definitively in larger, prospective clinical trials evaluating immunotherapy strategies for patients with metastatic anal cancer. The finding that patients with lymph node-only metastatic disease experience significantly more favorable survival to immunotherapy warrants further validation in the analysis of outcomes of future immunotherapy trials, which will hopefully evolve and continue to refine more personalized treatment approaches for patients with metastatic anal cancer.

## Figures and Tables

**Table 1:** Patient clinical and pathological features.

Patient Clinical and Pathological Features	Number (%)
Age (years, median; range)	59 (42–81)
Gender	
Female	84 (80)
Male	21 (20)
ECOG Performance Status	
0	30 (58)
1	20 (38)
2	2 (4)
Number of distant organs involved	
1	47 (45)
2	31 (30)
3	23 (22)
4+	4 (4)
Metastatic site	
Liver	49 (47)
Lung	40 (38)
Lymph node	28 (27)
Unresectable/locally recurrent	25 (24)
Bone	11 (11)
Peritoneum	8 (8)
Lines of systemic treatment	
1	105 (reference)
2	74 (71)
3	44 (42)
4	13 (12)
5	6 (6)
6	1 (1)
Metastatic disease timing	
Metachronous	64 (61)
Synchronous	41 (39)
Prior radiation	
Yes	77 (73)
No	28 (27)
HPV ^1^ tumor status	
HPV-positive	76 (97)
HPV-negative	2 (3)
HIV ^1^ status	
HIV-negative	81 (95)
HIV-positive	4 (5)

^1^ Abbreviations: HPV, human papillomavirus; HIV, human immunodeficiency virus.

**Table 2:** Systemic therapies selected across sequential lines of treatment for metastatic anal cancer.

Line of Therapy	1	2	3
Number	105	74	44
Chemotherapy (%)	**91 (87)**	**28 (38)**	**35 (80)**
Fluoropyrimidine + platinum	35	10	10
Taxane + platinum	51	15	18
Fluoropyrimidine + taxane + platinum	5	1	0
Topoisomerase inhibitor	0	2	2
**Immunotherapy (%)**	**14 (13)**	**46 (62)**	**9 (20)**
Anti-PD-(L)1 ^1^ antibody alone	9	35	7
Anti-PD-(L)1 ^1^ antibody combination	5	11	2

^1^ Abbreviations: Anti-PD-(L)1, anti-programmed death-(ligand)1.

## Data Availability

The data underlying this article are available in this article.

## Appendix A

**Table A1:** Comparison of clinical and pathological features between patients treated with immunotherapy and those not treated with immunotherapy.

Patient Clinical and Pathological Features	Patients Treated with Immunotherapy. Number (%)	Patients Not Treated with Immunotherapy. Number (%)	*p*-Value
Age (years, mean ± SD)	60.9 ± 8.4	61.1 ± 8.6	0.93
Gender			0.36
Female	57 (83)	27 (75)	
Male	12 (17)	9 (25)	
ECOG Performance Status			0.73
0	18 (26)	12 (33)	
1	11 (16)	9 (25)	
Number of Distant Organs Involved			0.26
1	28 (41)	20 (56)	
2	19 (28)	11 (31)	
3	18 (26)	5 (14)	
Metastatic Site			
Liver	31 (45)	18 (50)	0.62
Lung	29 (42)	12 (33)	0.39
Lymph Node	35 (51)	16 (44)	0.54
Unresectable/Locally Recurrent	9 (13)	2 (6)	0.25
Bone	8 (12)	3 (8)	0.61
Peritoneum	5 (7)	3 (8)	0.84
Metastatic Disease Timing			0.07
Metachronous	37 (54)	26 (72)	
Synchronous	32 (46)	10 (28)	
Prior Radiation			0.85
Yes	51 (74)	26 (72)	
No	18 (26)	10 (28)	
HPV Tumor Status			0.59
HPV-positive	52 (75)	24 (67)	
HPV-negative	1 (1)	1 (3)	

## Appendix C

**Table A2:** Associations between overall survival and clinicopathologic features for entire population of patients with metastatic anal cancer.

Clinicopathologic Feature	HR (95% CI)	*p*-Value
Age (greater vs. less than median)	0.86 (0.52–1.41)	0.54
Gender (male versus female)	1.48 (0.74–2.95)	0.21
ECOG Performance Status (0 versus 1)	0.44 (0.17–1.15)	0.06
Metachronous versus synchronous metastasis development	0.90 (0.54–1.50)	0.68
Prior radiotherapy (yes versus no)	0.75 (0.42–1.33)	0.29
Liver metastases (present versus absent)	1.25 (0.76–2.06)	0.38
Lung metastases (present versus absent)	0.85 (0.51–1.43)	0.55
Lymph node metastases (present versus absent)	1.36 (0.82–2.26)	0.22
Localized unresectable tumor (yes versus no)	0.88 (0.42–1.87)	0.75

**Table A3:** Associations between overall survival and clinical features for patients who received immunotherapy.

Clinicopathologic Feature	HR (95% CI)	*p*-Value
Age (greater vs. less than median)	0.77 (0.41–1.45)	0.42
Gender (male versus female)	1.32 (0.54–3.25)	0.47
ECOG Performance Status (0 versus 1)	0.20 (0.05–0.73)	0.01
Metachronous versus synchronous metastasis development	0.72 (0.38–1.35)	0.30
Prior radiotherapy (yes versus no)	0.57 (0.27–1.19)	0.13
Localized unresectable tumor (yes versus no)	0.74 (0.32–1.7)	0.74
